# Insights into Fluctuations of Structure of Proteins: Significance of Intermediary States in Regulating Biological Functions

**DOI:** 10.3390/polym14081539

**Published:** 2022-04-11

**Authors:** Zahoor Ahmad Parray, Mohammad Shahid, Asimul Islam

**Affiliations:** 1 Centre for Interdisciplinary Research in Basic Sciences, Jamia Millia Islamia, New Delhi 110025, India; zaparray@gmail.com; 2 Department of Chemistry, Indian Institute of Technology Delhi, IIT Campus, Hauz Khas, New Delhi 110016, India; 3 Department of Basic Medical Sciences, College of Medicine, Prince Sattam bin Abdulaziz University, Al Kharj 11942, Saudi Arabia; dr.shahid90@yahoo.com

**Keywords:** protein folding, intermediate states, biological functions, cellular conditions

## Abstract

Proteins are indispensable to cellular communication and metabolism. The structure on which cells and tissues are developed is deciphered from proteins. To perform functions, proteins fold into a three-dimensional structural design, which is specific and fundamentally determined by their characteristic sequence of amino acids. Few of them have structural versatility, allowing them to adapt their shape to the task at hand. The intermediate states appear momentarily, while protein folds from denatured (D) ⇔ native (N), which plays significant roles in cellular functions. Prolific effort needs to be taken in characterizing these intermediate species if detected during the folding process. Protein folds into its native structure through definite pathways, which involve a limited number of transitory intermediates. Intermediates may be essential in protein folding pathways and assembly in some cases, as well as misfolding and aggregation folding pathways. These intermediate states help to understand the machinery of proper folding in proteins. In this review article, we highlight the various intermediate states observed and characterized so far under in vitro conditions. Moreover, the role and significance of intermediates in regulating the biological function of cells are discussed clearly.

## 1. Introduction

The regulatory functions of proteins under in vitro complex systems and within the cell are well known. However, proteins that are unfolded or partially folded (intermediates) also play a significant role in different cellular processes and signaling events [1,2,3,4,5]. The role of such intermediates of protein folding has not been discussed in detail until now, and new findings are evolving to provide a fertile ground for considering the molecular mechanisms of biological processes [3,6]. The intermediate state of the protein with a native-like secondary structure but with an unstable or molten tertiary structure can be helpful in understanding pathways of protein folding [7,8]. Such intermediates offer new insights into the role of structural change in proteins within the cell, where transitional states of proteins can be imported and exported more efficiently via membranes than native forms of proteins [5,9]. The translocation of phospholipids between the two monolayers of a lipid bilayer of a cell membrane is carried out by a class of proteins called scramblases. It has been observed by researchers that scramblases are in a fully open state while they assume intermediate states and assist in the transport of ions [10]. These proteins that began as scramblases and were developed into pure ion channels, as a result of mutations, favor the intermediate type [10,11]. Such folding intermediates in amyloid disorders can help in understanding the protein folding and assembly routes, as well as those of misfolding and protein aggregation [12,13,14]. Folding intermediates help in the amyloid fibril formations, and these are resistant structures against dissociation and degradation and are long-lasting [15]. The amyloids are made up of β-strands arranged into sheets that lie perpendicular to the long fiber axis and have a central cross β-structure [16]. Exploring the protein folding research, one should know the phases that drive this protein folding. The first phase is spotlighted to understand the protein folding mechanisms and reveal the basic principles that govern transitions in folding processes. The general answers are provided to questions raised in protein folding in the primary phase. With evolution, innovative and significant questions arise, such as how proteins fold. What are the mechanisms of protein folding in the highly crowded condition of the cell, where proteins are surrounded by various biological molecules? These evolutionary questions on protein folding raised are answered by the second stage of the folding procedure. The primary phase is nearest and dearest to a romantic stage of the research; however, the secondary and final goal may not be directly valid to exploit and understand. This second stage is a more practical step of research, where the research field drives the purpose and allows the engineering of tools for progression to create significant science [17,18]. Understanding the functional intermediates that accompany the transitory protein’s journey to its native state could allow valid protein structure manipulation through protein design, which is an appropriate example of such engineering. There are so many studies where mutation in one amino acid [19,20,21,22], pH change, temperature disturbance, and cosolute (salts, polyols, crowders, etc.) presence lead native proteins to intermediate states [23,24,25,26,27,28,29,30,31,32,33,34]. This review addresses the elements for understanding the biologically significant mechanism of conformational changes, such as harmonic vibrations, structural distributions, and structural fluctuations.

## 2. Intermediate States of Proteins and Their Types

Almost all proteins fold via several partially structured intermediates. To comprehend the structure and structural characteristics of intermediates at the atomic level is often an argumentative content since these are characterized and monitored under an extreme environment of temperature, pH, and chemical denaturants. Besides, chemical modifications, site-directed mutagenesis (or point mutation), and cleavage of the covalent bond of natural proteins are several other routes that often lead to native and/or denatured-like intermediate structures include molten globule (MG) and premolten globule (PMG) states, respectively [20,21,22,31,35].

The molten globule (MG) states are partially unfolded structured forms enfolded with a prominent amount of a secondary structure but a largely chaotic tertiary structure [20,30,36,37,38]. These are compact and native-like structures of the protein considered to be general intermediate states in protein folding [20,39,40]. Because of their similarity to early kinetic intermediate states [36,41], MGs have been proposed as models for transient intermediates in protein folding. The first report of MG state was observed in 1981 [42], while the term MG state was coined in 1983 [41]. Furthermore, the MG states are classified into dry MGs (DMGs) and wet MGs (WMGs). In comparison with the native protein, the former state has slightly extended forms and dry interiors with more conformational flexibility [20,35,43], and the latter possesses hydrated cores with significantly reduced packing in similarity to the folded state [20,43]. Understanding protein folding problems necessitates a comprehensive insight into the characteristics of intermediate species and provides a clear proof of the importance of maintaining proper stoichiometry (as defined by the experimentally observed relative frequencies of amino acids) [44,45]. The following are the common structural features of MGs [30,46]: (i) the presence of a substantial amount of secondary structure (very comparable with that of the protein in native condition) is confirmed by far-UV circular dichroism (CD) and IR spectroscopy, but generally reduced stability of the constitutive hydrogen bonds as represented by proton exchange using ^1^H NMR [26,47]; (ii) the majority of the particular tertiary structure created by the close packing of side chains is missing as determined by near-UV CD and 2D nuclear magnetic resonance (NMR) [48]; (iii) the protein molecule compactness is with a radius of gyration 10–30% greater than that of the native state [49,50] or a hydrodynamic radius 15–16% greater than that of the native state [41,51]; and (iv) the solvent-exposed loosely packed hydrophobic patches (hydrophobic surface areas) are present due to which it acts sticky [52] and binds to the hydrophobic molecules, such as 8-anilino-1-naphthalenesulfonic acid (ANS) [27] and the Nile red [53].

**Figure 1:**
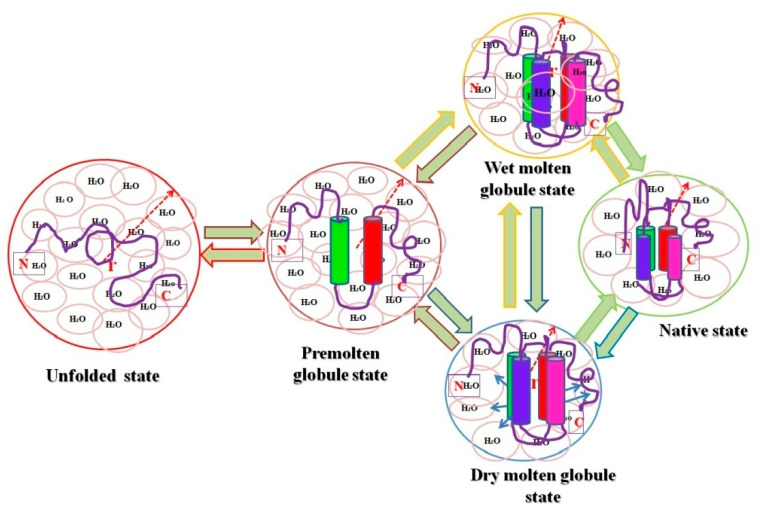
Pictorial representation of relative hydrodynamic volumes of different intermediate states of proteins. The figure shows an ordered secondary structure (cylinder shaped) and water molecules associated with each state (pink circles), and the arrows in dry molten globule (DMG) state represent an increase in the size towards the native state exclusive of water diffusion.

The premolten globule (PMG) state is less condensed than the MG and native states, but it is far more compacted than the unfolded state (random coil) [54]. Jeng and Englander [54] coined the term PMG in 1991. It is a partially unfolded form of the protein that is believed to be a general protein folding intermediate [36]. PMG states were discovered in several proteins during equilibrium intermediate studies, and are thus considered a fundamental thermodynamic state of the hierarchical protein folding processes [19,54,55,56]. For the past two decades, protein scientists have been intrigued by the PMG, not just because it provides insights into the classic three-stage unfolding process seen in many proteins, but also because it is comparable to the partly folded intermediate temporarily accumulating in the initial stages of folding. Many proteins’ PMG states have been successfully characterized under salt-induced denaturing conditions, such as LiCl and LiClO_3_ [19,57], by the interaction of many other divalent and trivalent metal ions, including Zn^2+^ [58], SDS-induced denaturing circumstances, and acidic pH [59,60], and more studies are there where PMG states of different proteins were successfully characterized (see Table 1) [28,33,54,56,59,61]. The PMG state is an equilibrium counterpart of the first kinetic folding intermediate formed within a few milliseconds (referred to as the burst-phase intermediate) and accumulates momentarily during refolding from a fully unfolded state [51,60]. The common structural characteristics of PMGs [62,63,64] are: (i) about 50% of the native secondary structure is present, which is revealed from far-UV CD and IR spectroscopy; (ii) no rigid tertiary structure is present as determined by near-UV CD; (iii) compactness (in terms of hydrodynamic volume) is roughly three times greater than that of the N state; and (iv) it shows almost five times weaker ANS binding than for the MG state. It is also widely understood that the protein molecule in the PMG state lacks a globular form, hinting that the PMG is most probably a squeezed, partially structured, and partially disordered conformation of a coil [61,65]. Finally, an all-or-none transition separates the PMG from the MG, which is an intramolecular analog of the first-order step transition [19,28,50,63]. These observations disclosed that both intermediate states (MG and PMG) characterize diverse thermodynamic states of globular proteins. A model has been proposed based on the above knowledge about folded, MG, PMG, and unfolded forms of proteins [35](see Figure 1). Recently, both the computational and spectroscopic approaches were exploited for the successful characterization of two intermediate states (MG and PMG) in myoglobin (Mb) induced by two different concentrations of PEG 4 kDa [66]. 

This study demonstrated that protein folding does not follow a single and unique pathway, but rather proceeds by various pathways through a folding funnel, similar to rain falling down a funnel, and there by foresees the energy landscape concept [66].

Recently, the metastable state of the protein was observed and characterized with unique structural properties that position the protein in the energy landscape’s local free energy minimum state [67]. The energy barrier that separates the energy minima of different conformations determines the brief, though finite, duration of a metastable state. The native-to-metastable structural transitions are driven by temporary or long-lived thermodynamic and kinetic variations of the protein molecules’ intrinsic connections. The representation of the structural and functional features of such metastable proteins is seen to be essential not only to understand the complexity of folding patterns, but also to explain the mechanisms of anomalous protein aggregation [67]. Consequently, identifying any periodic transient state of protein as a metastable state would be misleading. This conformation may be caused by inherent protein properties, such as heterogeneity of complexity areas, mutation, and folding anomaly, as well as environmental fluctuations, such as pH, ion concentrations, temperature, and pressure changes [67]. Proteins’ biological function depends on their structural dynamics [68,69]. To establish a coherent picture of the physics of intramolecular fluctuations and conformational changes, results from novel experimental methods should indeed be juxtaposed with those from previous studies. This review also addresses the elements for understanding the biologically significant mechanism of conformational changes, such as harmonic vibrations, structural distributions, and structural fluctuation.

### 2.1. Intermediate States Characterized under In Vitro Conditions

The intermediate states have been exerting a pull in recent times on scientists who put forth research on protein folding mechanisms to present hints for understanding the classical two-state and/or three-state unfolding methods. The first report on the MG state was observed in 1981, where the heat capacity function in the MG state of apo-α-lactalbumin was examined by a scanning microcalorimeter under physiological pH [42]. Observations of this study showed an enthalpy variation between the MG state and unfolded state (assumed) at neutral pH, which was observed to be almost zero, signifying that the MG state does not show sign any co-operative transition upon heating [42]. Preceding two decades, another third state, measured as a new thermodynamic state of the hierarchical protein folding process called the PMG, has drawn interest among scientists in the protein folding research field because it presents intimations to comprehend the classical three-phase mechanism in unfolding, observed in many proteins [19,28,31,33,54,55,56,59,61]. In vitro experiments have revealed that proteins can be guided to the MG state at acidic pH or high temperatures or in moderate doses of chemical denaturants [70,71]. Many proteins belonging to the structural class of all α or α + β have the majority of MG states characterized and classified in their folding/unfolding routes [72]. Interestingly, only a few studies of proteins found to be fit into all β-sheet categories, which were recognized as an MG state(s) [73,74,75]. Downhill folding mechanisms were intended to exist effectively for proteins with highly optimized native interactions under extremely stable conditions [76,77] or when constructive mutations take place [78]. Their study concluded that at least in metalloproteins, downhill folding can occur under a much greater choice of conditions and can be associated with a variety of other transitions [21]. According to the study, the bacterial zinc finger protein Ros87 has a bipartite folding/unfolding process in which a metal-binding intermediate converts to the native structure via a sensitive barrierless downhill transition. These intermediates were examined using DSC, CD, and NMR in a range of pH, temperature, and ionic strength parameters, showing that the downhill mechanism can be discovered under a considerably broader range of conditions and can be related to a variety of other transitions [21]. Table 1 provides details of the various intermediate state (PMG and MG) formations in various proteins under variable conditions, characterized by various types of techniques. The improvement in qualitative and quantitative understanding of the MG state can lead to a better understanding of the folding pathways and, as a result, could help solve the protein folding problem. Judy et al. [79] described in their recent review that the majority of investigations into protein MG states have been qualitative [79], and also showed that investigators utilize high-sensitivity calorimetry (differential scanning calorimetry and isothermal titration calorimetry) in endeavors to acquire quantitative understanding regarding MG states [79]. The computational findings on human α-lactalbumin carried out by Paci et al. [80] confirms that MG state unfolding is not a cooperative process, on account of the suggestion that the structural elements of the protein do not unfold simultaneously [80].

**Table 1:** List of various intermediate states of proteins characterized under in vitro conditions using various techniques.

S. No.	Protein	State Type	Conditions	Techniques Exploited	Ref.
1.	Apo-α-lactalbumin	MG	At neutral pH (7.6) and low ionic strength	Scanning microcalorimeter	[42]
2.	Apo-α-lactalbumin	MG	The transition around 25–30 °C at pH 8.1 in the presence of 10 mM borate and 1 mM EGTA	Intrinsic protein fluorescence, circular dichroism (CD), and differential scanning microcalorimetry (DSC)	[7]
3.	α-Lactalbumin	MG	Guanidinium chloride (GdmCl)-induced (1.8 M) and 1 mM Ca^2+^ at 4.5 °C, pH 7.0 in the presence of 0.05 M sodium chloride (NaCl) and 0.05 M sodium cacodylate	Circular dichroism (CD) spectroscopy and nuclear magnetic resonance	[81]
4.	Myoglobin	MG	PEG 10 (300 mg mL^−1^) at pH 7.0 and 25 °C	Absorption, fluorescence and CD spectroscopy, ANS binding, dynamic light scattering (DLS), FTIR, isothermal titration calorimetry (ITC)	[30]
5.	Myoglobin	MG	Ficoll 70 (300 mg mL^−1^) at pH 7.0 and 25 °C	CD spectroscopy, intrinsic and ANS fluorescence, DLS, and ITC measurements	[29]
6.	Myoglobin	PMG	PEG 400 (320 mg mL^−1^) at pH 7.0 and 25 °C	CD spectroscopy, intrinsic and ANS fluorescence, DLS, and ITC measurements	[33]
6.	Myoglobin	MG	Around 300 K (26.85°C) −500 K (226.85 °C), apo-Mb like intermediate state for 2–9 ns (nanoseconds) at pH 7.0	In silico method (i.e., molecular dynamic (MD) simulations)	[82]
5.	Myoglobin	MG	Cobalt(III) induced (10 μM) in 0.01 M sodium phosphate buffer solution at pH 6.5 and 25 °C	UV–VIS absorption and CD spectroscopy	[83]
7.	Myoglobin	MG	4% (*/v*) HFIP (aqueous hexafluoroisopropanol) at pH 4.0	CD spectroscopy	[84]
9.	Apo-myoglobin (Apo-Mb)	MG	Site mutagenesis studies at pH 7.0 and pH 3.0	Fluorescence and CD spectroscopy	[85]
10.	Apo-myoglobin (mutants)	MG	Mutation in apo-Mb (S108L, F123W, F123G, and A130S) in the presence of 10 mM sodium acetate buffer at 0 °C around acidic pH	Circular dichroism (CD) spectroscopy, nuclear magnetic resonance	[26]
11.	Apo-myoglobin	MG	Acid-induced unfolding at 0 °C, 2 mM sodium citrate in the presence of various urea concentrations	Circular dichroism (CD) spectroscopy, nuclear magnetic resonance	[86]
12.	Apo-myoglobin	PMG	In the presence of different anions (100 mM trifluoroacetate) at pH 2.0 and 25 °C	Tryptophan and ANS binding fluorescence, CD spectroscopy, FTIR, small-angle X-ray scattering, and DLS	[87]
16.	Cytochrome *c*	MG	PEG 400 induced at pH 7.0 and 25 °C	Absorption, fluorescence and CD spectroscopy, DLS, and ITC measurements	[88]
17.	Cytochrome *c*	MG	Induced by LiClO_4_ (1.85–3.3 M) at pH 6.0 and 25 °C	CD spectroscopy, intrinsic and ANS fluorescence, and DLS and intrinsic viscosity measurements	[32]
18.	Yeast iso-1-cytochrome *c* and its deletants	PMG	Induced by LiCl at pH 6.5 at 25 °C	Absorption, fluorescence, and CD spectroscopy and DLS measurements	[55]
19.	Cytochrome *c* (mutant Leu94Gly)	PMG	Induced by LiCl at pH 6.5 at 25 °C	Tryptophan fluorescence, ANS binding, CD spectroscopy, and DLS measurements	[57]
20.	Cytochrome *c*	PMG	NaCl-induced L94G mutation at pH 2 and 25 °C	CD spectroscopy, intrinsic and ANS fluorescence, and DLS measurements	[19]
13.	Cytochrome *c*	MG	Mutation of Leu94Gly at pH 6.0 and 25 °C	CD spectroscopy, intrinsic and ANS fluorescence, and DLS measurements	[19]
14.	Cytochrome *c*	MG	Leu94 by Val and Ile, at pH 6.0 and 25 °C	Intrinsic fluorescence and CD spectroscopy and differential scanning microcalorimetry (DSC)	[22]
15.	Cytochrome *c*	MG	Leu94 by Phe at pH 6.0 and 25 °C	Intrinsic fluorescence and CD spectroscopy, ANS binding, and DSC measurements	[89]
20.	Cytochrome *c*	PMG	NaCl-induced L94G mutation at pH 2 and 25 °C	CD spectroscopy, intrinsic and ANS fluorescence, and DLS measurements	[19]
21.	Cytochrome *c*	MG	Polyol-induced (ethylene glycol, glycerol, erythritol, xylitol, sorbitol, and inositol) at pH 2.0	Circular dichroism (CD) spectroscopy, partial specific volume, adiabatic compressibility, and DSC	[90]
22.	Yeast iso-1-cytochrome *c* and its deletants	MG	In the presence of 0.33 M Na_2_SO_4_ at pH 2.1	Absorption, fluorescence, and CD spectroscopy and DLS measurements	[55]
23.	Cytochrome *c*	MG	Sodium perchlorate stabilized at pH 1.8	Isothermal titration calorimetry, CD spectroscopy and DSC	[91]
24.	Sheep serum albumin	MG	GdmCl (2.38 M)-induced denaturation and urea (4.2–4.7 M)-induced denaturationin10 mM Tris-HCl buffer at pH 7.4 and 25 °C	Intrinsic and ANS binding fluorescence, CD spectroscopy, and DLS measurements	[92]
25.	Bovine carbonic anhydrase B	PMG	At 4 °C in 0.1 M sodium phosphate buffer (pH 6.8) in the presence of GdmCl concentrations	Tryptophan and ANS binding fluorescence, CD spectroscopy, size-exclusion chromatography (SEC-FPLC)	[28]
26.	GlutaminyltRNA synthetase (GlnRS)	PMG	Induced by 0.25 M potassium L-glutamate (natural osmolyte) in the presence of urea, 0.1 M Tris-HCl buffer of pH 7.5 at 25 °C	Tryptophan and ANS binding fluorescence, CD spectroscopy, and DLS measurements	[93]
27.	Recombinant human Stefan B	MG-states (G, A, and T)	G-state:in the presence of 1.7 M GdmCl (pH 8, 25 °C), A-state: at pH 4 (0.6 M GdmHCl, 25 °C), and T-state: formed above 68 °C	UV–VIS absorption and CD spectroscopy	[94]
28.	Pancreatic trypsin inhibitor (BPTI)	MG	Five MD simulations (lasting up to 550 ps) were performed: native BPTI at 298 K (25 °C) and 423 K (150 °C); reduced BPTI at 298 K (25 °C), 423 K (150 °C), and 498 K (225 °C); all simulations were carried out in a bath of water molecules with mobile counter ions	MD simulations	[95]
29.	Casein	PMG and MG	Physiological conditions (around pH 7)	Raman spectroscopy, FTIR, DLS measurements, and molecular kinetics	[96]
30.	Lysozyme	MG	At pH 2.0	Hydrogen exchange measurements, NMR, molecular graphics by MolScript	[97]
31.	Ribonuclease A	MG	At low pH (1.5—3.8) and 65 °C	Quenched flow methods, CD spectroscopy, pulsed H/D-exchange, and 2 D ^1^H NMR spectroscopy	[98]
32.	Ubiquitin	MG	At pH 2.0 and 25 °C in the presence of 60% methanol and 40% water	Pulsed H/D-exchange, NMR	[99]
33.	Zinc finger protein Ros87	Metal-binding intermediate	At pH 6.5 and temperature range of 25—99 °C (observed at 70 °C by NMR)	CD, DSC, NMR	[21]
34.	Apoflavodoxin	Thermal intermediate	At pH 7.0 and 95 °C	Atomistic multi-microsecond-scale molecular dynamics (MD) simulations, small-angle X-ray scattering, near-UV absorbance spectra	[100]
35.	Bovine serum albumin (BSA)	MG	In the presence of ANS and pyrene at pH 4.2	ANS fluorescence (supplemented by CD spectroscopy, light scattering, and analytical centrifugation)	[101]
36.	Staphylococcal nuclease (SNase)	Three different partially folded intermediates (A states: A_1_, A_2_, and A_3_)	Induced by anions: A states are stabilized by: (1) A_1_: induced by chloride (600 mM) or sulfate (100 mM): 50% native-like structure (2) A_2_: Induced by trifluoroacetate (300 mM): 70% native-like structure (3) A_3_: trichloroacetate (50 mM): fully native-like structure	CD and small-angle X-ray scattering (SAXS)	[102]

### 2.2. Significance of Intermediary States under In Vivo Conditions

Taking benefits from the protein folding (new wing) (i.e., intermediate states in the cellular conditions) upholds cellular protein homeostasis (proteostasis), which is critical for cell function and development [103,104]. Besides, the folding process—these intermediates assist in many genetic illnesses [8,105,106]. Proteostasis is governed in cellular conditions by networks of protein complexes that include the translation machinery [107,108], proteases [109,110], ubiquitin–proteasome system (UPS) [111], secretory pathways [112,113], autophagic machinery [114], and molecular chaperones [3], which have a significant role in protein homeostasis. To illustrate, a non-native compact type of cyt *c* is implicated in programmed cell death (induces apoptosis), after which the protein is released from the mitochondrion; non-native forms of the protein are also associated in several of the amyloid-related illnesses [8]. Characterizing the heterogeneity present within the process of folding and unfolding proteins, intermediate states are vital to understanding intermediates and defining their boundaries. The cell intermediate states can be defined as attractors on a potential landscape [1,34,115].

The intermediates not only help to decipher the enormously complex troubles in protein folding, although this also reveals new insight into the importance of structural changes in proteins within cells, whereas protein intermediates can be imported and exported more easily through membranes than native proteins [2,3,116]. The native ⇔ molten globule transition is also considered because the conversion of a protein’s native state to a condensed intermediate structure might occasionally allow it to perform different physiological activities inside the cell [8]. A non-native compact conformation of cyt *c*, for example, is linked to programmed cell death (apoptosis), whereupon the protein is released from the mitochondrion; non-native forms of the protein are also linked to various amyloid-related diseases [8]. Nuclear genes code for the majority of mitochondrial proteins, which are formed on cytoplasmic ribosomes and transferred into mitochondrial subcompartments [2,117]. To preserve the integrity of protein function in cellular compartments, protein sorting and transport through the lipid membrane of the mitochondrion is desired without intervening with the organelle’s integrity or functions. To understand this to a greater extent, molecular specificity and targeting of mitochondrial preprotein mechanisms and postproteins after import–export via an inner membrane and outer membrane facilitates recognition or identification and is characterized by cellular signaling [2,117]. The presence of these intermediate structures of protein has a significant role in transport via membranes in cellular conditions. The purpose of a set of proteins identified as heat-shock proteins or molecular chaperones located both outside and within the mitochondrion are intimately connected to the unfolding and folding of proteins during transmembrane movement. Investigating the folding of polypeptides in the mitochondrial matrix has provided new and unique findings into general protein folding pathways supported by folding factors [2,117]. Folding and misfolding of proteins in the human membrane help in the resolution of problems related to health and diseases [118]. The new perspective that links membrane protein folding energetics with the degree of complexity of biological systems is recognized via intermediates that play an essential role in the import–export of native protein via membranes and can easily interact with the drug to cure diseases. These advancements in the production of therapeutics and precision medicine are influenced by these intermediate structures in cells [118,119]. We know that from Anfinsen’s experiments [120], which provided how proteins choose their structural elements from denatured conformations and each fraction competes for renaturation to native state [118,121]. The complexity of protein folding makes it difficult to comprehend and even describe the process. Much of this heterogeneity can be described and understood using a statistical approach to the energetics of protein structure (i.e., the energy landscape) [76]. The statistical energy landscape strategy describes why and when particular folding pathways emerge in some proteins, and also how to spot the difference between folding mechanisms that are universal to all sequences and those that are specific to individual sequences. This method also provides fresh quantitative ideas in understanding protein folding thermodynamics and kinetic studies and simulations [68,76].

In the past, intermediates were thought to be necessary stepping stones that helped a protein go through the folding process to its native state. However, the discovery of multiple tiny proteins that fold rapidly without intermediates, as well as the introduction of new conceptual frameworks from computational research, led to the notion that intermediates can operate as energy sinks or dynamical traps, resulting in less efficient folding [5]. Proteins’ biological function depends on their structural dynamics [68]. To establish a coherent picture of the physics of intramolecular fluctuations and conformational changes, results from novel experimental methods should indeed be juxtaposed with those from previous studies [68]. Besides nuclear magnetic resonance (NMR) and spectroscopy studies, computational methods have all been used to uncover the activation route of proteins to study protein folding and intermediates [5,52,66,95,108,122,123,124,125]. MD simulations of MG and native states of pancreatic trypsin inhibitor (BPTI) were observed lasting up to 550 picoseconds (ps), at 298 K (25 °C) and 423 K (150 °C), and its reduced form was also studied at 298 K (25 °C), 423 K (150 °C), and 498 K (225 °C). The polypeptide segments that were determined to be the most flexible in the MD simulations were closely related to those that showed variations between the crystal and solution structures of BPTI [95]. Additionally, the G protein-coupled receptor activation pathway reveals conformational intermediates as potential targets for allosteric drug design [123,126]. To investigate the conformational landscape of the angiotensin II (AngII) type 1 receptor (AT1 receptor),a prototypical class A GPCR activation, the researchers used a cumulative computational and experimental framework that included comprehensive molecular dynamics simulations, Markov state models, site-directed mutagenesis, and conformational biosensors [123,126]. The evidence points to a synergistic AT1 receptor activation transition mechanism. The activation pathway has a critical intermediate state that has a cryptic binding site within the intracellular area of the receptor [123]. Mechanistic and structural insights into the conformational shifts that underpin the Ras deactivation pathway could lead to the development of specific treatments for Ras-driven cancers [125]. However, atomistic molecular dynamics (MD) simulations have yet to perfectly represent a large-scale conformational shift. For revealing the conformational landscape of the Ras deactivation route, a computational strategy that incorporates a transition pathway creation tool, extensive MD simulations, and Markov state model analysis was used by researchers [125]. From the study, they suggested that a gradual (stepwise) deactivation process for Ras hydrolysis, as well as the identification of numerous critical conformational substrates along the way, occurs [125]. Using an atomic force microscope to fold single ubiquitin molecules revealed a dynamic long-lived intermediate with nanometer-scale end-to-end distance fluctuations throughout a surprisingly extensive folding pathway [69]. Molecular dynamics refolding simulations of unfolded ubiquitin under constant tension were used to investigate the structure of this intermediate at the atomic level, as well as the driving forces that cause the observed fluctuations [69]. The researchers observed a very dynamic, broad ensemble of conformations with a partial and continuously changing secondary structure and side chain interactions after an initial rapid collapse and found this ensemble with features like that of a molten globule [69]. The pieces of evidence have also shown that conformational changes between active and inactive states of biomolecules can reveal two types of binding sites (cryptic or hidden) in protein kinases, such c-src [127], PKA [128], and PKB/AKT1 [129] structural substrates.

To know the mechanism of aggregation in the proteins in cellular conditions where the role of intermediate state(s) also exists, it needs best examples of proteins, such as the fibril formation observed in α-synuclein and other related proteins in the earlier stages involving partial folding of the protein(s) [5,104,130,131,132,133]. These partially folded structures change into the highly fibrillation-prone structure, which has no tertiary structure present, and half of the secondary structure is lost. These intermediates represent a key in the fibrillation pathway and have characteristics and conformation like that of PMG [51,132,134]. Few factors which includes non-polar molecules (preferentially bound to these partially intermediates), point mutations, high proton concentration cations, and oxidative damages leads these partially folded conformations into fibril development [78,131]. The presence of definite aggregates of α-synuclein enhances toxicity in different ways in cellular processes [130]. The structural mechanisms, by which intermediates promote fibrillar aggregation, have remained largely unexplored. Protein-folding intermediates linked to the development of amyloid fibrils are involved in neurodegenerative diseases [132,135,136]. The structure of a low-populated, on-pathway folding intermediate of the A39V/N53P/V55L (A, Ala; V, Val; N, Asn; P, Pro; L, Leu) Fyn SH3 domain was determined using relaxation dispersion nuclear magnetic resonance spectroscopy [132]. In this intermediate, the carboxyl terminus remained unstructured, exposing the aggregation-prone amino-terminal β-strand. The structures elaborate the non-native interactions that maintain an aggregation-prone intermediate under native conditions, as well as how such an intermediate can disrupt folding and induce fibrillation [132].

Advances in the protein chemistry research field develop an understanding that intermediates may occur during protein folding and unfolding to help in understanding proteins that occur in a variety of structure forms (α, β,and γ). Therefore, many proteins turn up natively unfolded, intrinsically disordered, or unstructured under physiological conditions. Casein proteins present in the milk are a diverse group of proteins exhibiting a strong tendency to associate with themselves and with each other. These features help in generating the protein’s different structures and oligomeric species [137]. The casein proteins are not random coils but are present in different newly described intermediate states with variable properties. Because of this fact, the casein structure is still being disputed and has been explanatory on how these intermediate structures fit the definite protein. These new research systems have strengthened our understanding of its properties, allowing us to explore new possibilities. The protein is more than just a dietary protein; its structural intermediates and properties promise different and novel uses in research, pharmaceuticals, and functional foods. If these concepts are applied to casein fractions, it may be feasible to produce effective food products having nutraceutical or nanotechnological utilization [119,138,139].

The point mutations in several proteins lead to genetic diseases [140,141]. These mutations have caused proteins to be misplaced in a cell, resulting in their loss of function [116], therefore influencing protein trafficking associated with some human genetic diseases. It is interesting as a point of view to be considered that the MG or comparable structural states of protein molecules may be involved there [70,108,119,142]. In vitro studies have shown that site-directed mutagenesis leads to the formation of protein intermediates [22,89]. Some physiological processes, such as protein recognition by chaperones, secretion of protein ligands, and protein translocation through bio-membranes, have already been suggested to involve the MG states [70,143]. There are very strong facts and shreds of evidence that confirm that non-native or denatured conformational states of the proteins help in their translocation via membranes. These states are internally mobile and compact but adequately extended to include water. These molten globule states are thought to be good runners for protein translocation through biological membranes [144].

**Figure 2:**
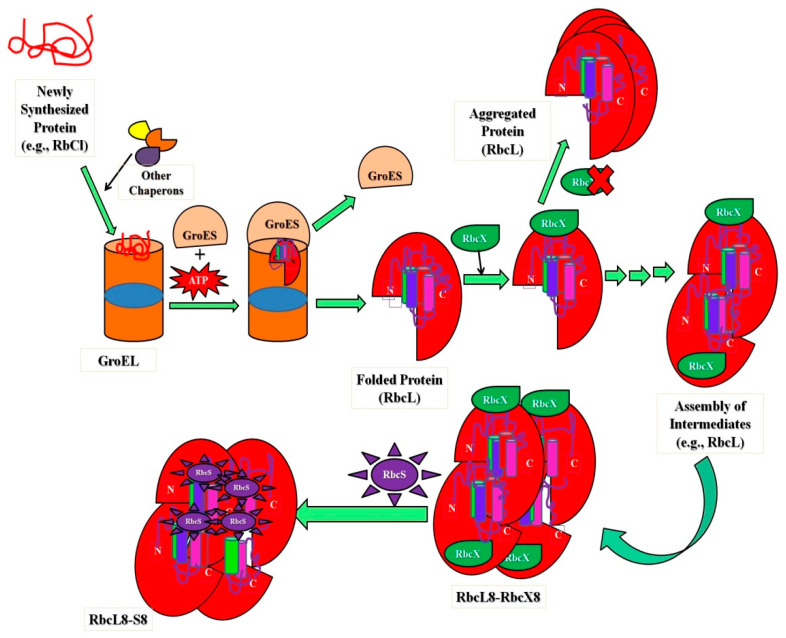
Schematic representation showing the importance of intermediates in folding and assembly of L8S8 Rubisco (cyanobacterial) mediated by GroEL/GroES and RbcX.

The ribulose-bis-phosphate carboxylase/oxygenase, Rubisco (an abundant protein on earth), has greater kinetic facets in plants to enhance photosynthesis quality, resulting in species with high nitrogen and water-use efficiencies. This protein improves crop improvement and can provide relief from the CO_2_ increase caused by anthropogenic activities that lead to global climate changes. Type I Rubisco is a highly conserved hexa-decameric complex found in cyanobacteria, algae, and plants. It consists of eight large subunits with ~50 kDa molecular mass and eight small subunits with ~15 kDa molecular mass. Another kind of bacterial Rubisco (Type II) is a dimer of large subunits that folds and assembles spontaneously in a GroEL-mediated reaction [145,146]. Whether GroEL/GroES was co-overexpressed or not, the expression of Type I Rubisco (from cyanobacteria) in *E. coli* did not result in the formation of soluble protein, in comparison with the bacterial process. The investigations noticed that in cyanobacteria, the Rubisco operon holds an ORF (open reading frame) for a protein called RbcX. Apart from the genes for the Rubisco subunits, there are genes for the small and large Rubisco subunits (RbcS and RbcL, correspondingly). The existence of the protein (RbcX) was very less renowned before, but researchers have developed methods in *E. coli* and express Rubisco upon coexpression of RbcX. A Rubisco-specific chaperone could thus be a crucial step in allowing efficient folding of imported Rubiscos in both prokaryotic and plant systems [105,147]. Figure 2 shows the significant role of intermediate assembly in the folding and assembly of L8S8 Rubisco mediated by GroEL/GroES and RbcX. The study showed that folding process includes steps, (i) the substrate bound (primary state) to the chaperonin complex, (ii) structural characterization of intermediate states kinetically trapped and accumulated throughout the folding route, and (iii) kinetic measurements during the process (unfolded ⇔ intermediate ⇔ native state conversion) [145].

In addition, the researchers observed that folding intermediates provide approaches to differences in immunoglobulin amyloidogenicity and thus can shape the folding landscape positively to favor either folding or misfolding [3,111,134,148]. The researchers used an antibody domain’s intrinsically slow folding process to define its essential folding intermediate [134]. They were able to trap the intermediate in equilibrium and identify it at atomic resolution using a single-point mutation. It is also worth noting that intermediate has the simple β-barrel topology; however, a few strands were observed to be distorted [134]. Unexpectedly, the presence of two short-strand-connecting helices in the constant region of antibody domains suggests that a native structure is fully present in the intermediate, which was then used as a framework for subsequent strands [134]. Transplanting these conserved stands of helices into β 2-microglobulin (homologous member of the same superfamily) considerably showed a reduction in its amyloidogenicity [134]. As a result, a high level of local structuring intermediates through protein folding can have a considerable effect on the folding landscape which favored vigorous folding against negative misfolding. In addition, throughout evolution, the small differences acquired amid members of the identical protein superfamily can evade pathogenic misfolding reaction and identical protein topology conservation [134].

As it is a known fact that transferable agents called prions cause spongiform encephalopathies (TSEs) in animals as well as humans. They are made up of PrPSc, the infectious isomer of PrPC, and the cellular prion protein [149]. The conversion and propensity of the protein commence alternative folds, which are liable for the species-specific transmission of the disease. Kachel et al. defined and confirmed the structural stages of the human prion protein (hu PrP) [149] by using a hydrostatic pressure (up to 200 MPa) and two-dimensional NMR spectroscopy in combination. They recognized folding intermediates that were stabilized by pressure of the human prion protein. They observed that the β1/α1-loopand the solvent-exposed side of α3 are the strongest regions reflecting the transition to the intermediate states [149]. Their findings showed that the loop between β-strand 1 and α-helix 1 (residues 139–141) was the most pressure-sensitive region (intermediate I_1_), and may be the first gateway for the infectious moiety to transform the cellular protein [149].

Therefore, folding intermediates are essential in determining protein folding parameters, understanding protein folding mechanisms, conservation of protein topology, cellular transport regulation, structural maintenance, and avoidance of protein misfolding. These elements are also better for understanding the biologically significant mechanism of conformational changes, such as structural distributions, harmonic vibrations, and structural fluctuations [68,69]. Therefore, it may not be wrong to say that intermediates are vibrant and vigorous elements of cellular architecture.

## 3. Conclusions

An intermediate state of proteins is a transitory state in the protein folding that exists inside the living organism. These intermediate states can be induced by changing the environment ofthe native protein (pH change, chemical induced, temperature induced, and so on) and can be characterized under an in vitro system. These intermediates are significant with their roles including cellular transport, structural maintenance, the prevention of protein misfolding, and conservation of protein topology. They are transient and perform their regulation and maintain the integrity of cellular functions. Therefore, folding intermediates play a central role in determining protein folding and comprehending the protein folding mechanisms that help to understand the structural conformation of proteins under an in vivo system.

## Data Availability

Not applicable.

## Declarations

Abbreviations: 8-Anilinonaphthalene-1-sulfonic acid (ANS), bovine pancreatic trypsin inhibitor (BPTI), bovine serum albumin (BSA), circular dichroism (CD), diffraction scanning calorimetry (DSC), dry molten globule (DMG), dynamic light scattering (DLS), Fourier-transform infrared (FTIR), human prion protein (hu PrP), isothermal titration calorimetry (ITC), molecular dynamics (MD), molten globule (MG), nuclear magnetic resonance (NMR), premolten globule (PMG), ribulosebisphosphate carboxylase/oxygenase, (Rubisco), small-angle X-ray scattering (SAXS), sodium dodecyl sulfate (SDS), staphylococcal nuclease (SNase), and wet molten globule (WMG).
